# A Bee Evolutionary Guiding Nondominated Sorting Genetic Algorithm II for Multiobjective Flexible Job-Shop Scheduling

**DOI:** 10.1155/2017/5232518

**Published:** 2017-03-28

**Authors:** Qianwang Deng, Guiliang Gong, Xuran Gong, Like Zhang, Wei Liu, Qinghua Ren

**Affiliations:** State Key Laboratory of Advanced Design and Manufacturing for Vehicle Body, Hunan University, Changsha 410082, China

## Abstract

Flexible job-shop scheduling problem (FJSP) is an NP-hard puzzle which inherits the job-shop scheduling problem (JSP) characteristics. This paper presents a bee evolutionary guiding nondominated sorting genetic algorithm II (BEG-NSGA-II) for multiobjective FJSP (MO-FJSP) with the objectives to minimize the maximal completion time, the workload of the most loaded machine, and the total workload of all machines. It adopts a two-stage optimization mechanism during the optimizing process. In the first stage, the NSGA-II algorithm with *T* iteration times is first used to obtain the initial population *N*, in which a bee evolutionary guiding scheme is presented to exploit the solution space extensively. In the second stage, the NSGA-II algorithm with* GEN* iteration times is used again to obtain the Pareto-optimal solutions. In order to enhance the searching ability and avoid the premature convergence, an updating mechanism is employed in this stage. More specifically, its population consists of three parts, and each of them changes with the iteration times. What is more, numerical simulations are carried out which are based on some published benchmark instances. Finally, the effectiveness of the proposed BEG-NSGA-II algorithm is shown by comparing the experimental results and the results of some well-known algorithms already existed.

## 1. Introduction

As a part of production scheduling and combinatorial optimization problems, job-shop scheduling problem (JSP) attracts more and more researchers from all walks of life (e.g., mechanical engineering, mathematics, and computer software engineering) in the recent decades [1–5].

Flexible job-shop scheduling problem (FJSP) inherits the characteristics of the JSP, in which each operation is allowed to be processed by any machine in a given set rather than one specified machine, and it has been proved that the FJSP is strong NP-hard [6]. FJSP consists of two subproblems: one is the routing subproblem that each operation is assigned to one machine of a set of machines, and the other is the scheduling subproblem that a feasible schedule is obtained by sequencing the assigned operations on all machines. Therefore the FJSP is more difficult to be solved than the classical JSP because of its need to determine the assignment of operations in related machines [7].

The FJSP is firstly addressed by Brucker and Schlie [8]. And they presented a polynomial algorithm with only two jobs and identical machines. Brandimarte [9] proposed a hybrid tabu search (TS) algorithm which was based on decomposition to solve the FJSP. Dauzère-Pérès and Paulli [10] provided a TS algorithm which was based on the integrated approach and developed a new neighbourhood function [11] for the FJSP in terms of solution quality and computation time. Gao et al. [12] proposed a hybrid genetic algorithm to solve the FJSP with nonfixed availability constraints. And in order to enhance the inheritability, this genetic algorithm uses an innovative representation method and applies genetic operations to phenotype space. Saidi-Mehrabad and Fattahi [13] developed a TS algorithm that took the operation sequences and sequence-dependent setups into consideration to solve the FJSP. A genetic algorithm (GA) combined with a variable neighbourhood search (VNS) was presented by Gao et al. [14], and a GA with different strategies was proposed by Pezzella et al. [15]. Yazdani et al. [16] developed a parallel VNS algorithm based on the application of multiple independent searches which increased the exploration of search space. Xing et al. [17] put forth a knowledge-based ant colony optimization algorithm. Recently, a novel artificial bee colony (ABC) algorithm [18] and a discrete harmony search (DHS) algorithm [19] were brought forward to solve the FJSP.

As is shown above, the single-objective optimization of FJSP (SO-FJSP) has been extensively studied, which generally minimizes the makespan that is the time required to complete all jobs. However, many industries (e.g., aircraft, semiconductors manufacturing, and electronics) have trade-offs in their scheduling problems in which multiple objectives need to be considered to optimize the overall performance of the system. Therefore, the MO-FJSP may be closer to the realistic production environments and needs to be further studied. In recent years, the MO-FJSP has captured more and more interests of numerous domain researchers, and a great many of algorithms have been presented [20]. Compared with SO-FJSP, MO-FJSP has two problems to be dealt with: incommensurability between objectives and contradiction between objectives (i.e., optimizing a single-objective generally results in deterioration of another objective) [13].

For the MO-FJSP, Schaffer [21] provided a genetic algorithm with vector evaluated. Jurisch [22] presented a branch-and-bound algorithm and some heuristic algorithms. By combining the VNS with particle swarm optimization (PSO), Liu et al. [23] presented a hybrid metaheuristic to solve the MO-FJSP. A new genetic algorithm (GA) which hybridized with a bottleneck shifting procedure was developed by Gao et al. [24] to solve the MO-FJSP. Zhang et al. [25] embedded tabu search (TS) in PSO as a local search to deal with the MO-FJSP. Xing et al. [26] advanced an efficient search method for the MO-FJSP. González-Rodríguez et al. [27] proposed a generic multiobjective model which was based on lexicographical minimization of expected values for FJSP. By introducing several metrics of the multiobjective evaluation in the MO-FJSP literature, Rahmati et al. [28] adopted two multiobjective evolutionary algorithms for the MO-FJSP.

Recently, some studies based on the Pareto dominance relation have been used to solve the MO-FJSP and they are more desirable when compared with the prior linear weighted summation ones [29]. The nondominated sorting genetic algorithm (NSGA) [30] was one of the first methods used to solve the multiobjective problem. Kacem et al. [31] brought up a Pareto approach based on the hybridization of fuzzy logic (FL) and evolutionary algorithms to solve the MO-FJSP. Ho and Tay [32] integrated a guiding local search procedure and an elitism memory mechanism into the evolutionary algorithm to solve the MO-FJSP.

Deb et al. [33] came up with a nondominated sorting-based multiobjective evolutionary algorithm (MOEA), called nondominated sorting genetic algorithm II (NSGA-II). Wang et al. [34] proposed a multiobjective GA based on immune and entropy principle for the MO-FJSP. By employing simulated annealing (SA) algorithm as a local search process, Frutos et al. [35] introduced a memetic algorithm (MA) based on the NSGA-II. Wang et al. [36] presented an effective Pareto-based estimation of distribution algorithm (P-EDA), in which various strategies are integrated to maintain quality and diversity of solutions. Rohaninejad et al. [37] advanced an MO-FJSP problem with machines capacity constraints to minimize the makespan and overtime costs of machines. Kaplanoğlu [38] put forth an object-oriented (OO) approach combined with SA optimization algorithm to solve the MO-FJSP, in which a two-string encoding scheme was used to express this problem.

Our review of the above literatures reveals that the NSGA-II algorithm has been widely used to solve the MO-FJSP for its advantages such as high efficiency to optimize the complex problems and the ability to gain widespread Pareto-optimal solutions. And the algorithms with a two-stage optimization scheme have been also widely studied to solve the MO-FJSP for it could fully tap the optimization potentials of various metaheuristic algorithms. However, we found that the NSGA-II algorithm has the disadvantage of premature convergence to local solution and the algorithms with a two-stage optimization scheme have the disadvantages of being unable to gain stable and high quality initial population in the first stage. Hence, in this paper, we propose a bee evolutionary guiding NSGA-II (BEG-NSGA-II) with a two-stage optimization scheme to solve the MO-FJSP, which aims to fully play the respective advantages of NSGA-II algorithm and the algorithms with a two-stage optimization scheme and to overcome the disadvantages of them. In the first stage, the NSGA-II algorithm with *T* iteration times is first used to obtain the initial population *N* which consists of three parts changing with the iteration times. In order to extensively exploit the solution space, an effective local search operator is invented in this stage. In the second stage, the NSGA-II algorithm with* GEN* iteration times is used to obtain the Pareto-optimal solutions. In order to enhance the searching ability and avoid the premature convergence, an updating mechanism and some useful genetic operators were employed in this stage. Four famous benchmarks that include 53 open problems of FJSP are chosen to estimate the performance of the proposed algorithm. Moreover, by comparing the results of our algorithm and some existing well-known algorithms, the virtues of our algorithm can be clearly demonstrated.

The rest of this paper is organized as follows. The definition and formalization of the MO-FJSP are given in the next section. In Section 3, NSGA-II is briefly introduced, and then the overview and implementation details of the proposed BEG-NSGA-II are presented, respectively. Afterwards, Experimental Studies and Discussions are made in Section 4. Finally, Conclusions and Future Studies are described in Section 5.

## 2. Problem Definition

The FJSP is commonly defined as follows. There is a set of *n* jobs (*J*_*i*_, *i* ∈ {1,2,…, *n*}) and a set of *m* machines (*M*_*k*_, *k* ∈ {1,2,…, *m*}). One or more operation(s) (*O*_*ij*_, *j* ∈ {1,2,…, *n*_*i*_}, *n*_*i*_ is the total number of operations for job *J*_*i*_) is/are allowed to be processed by one machine of *M*_*ij*_, which consists of a set of machines of the *j*th operations for job *J*_*i*_. *P*_*ijk*_ denotes the processing time of the *j*th operation for job *J*_*i*_, which is processed by machine *k*. Generally, the FJSP consists of two subproblems: the routing subproblem of assigning each operation to a machine among a set of machines available and the scheduling subproblem of sequencing the assigned operations on all machines to obtain a feasible schedule for optimizing a certain objective function [39, 40].

One classical 4 × 5 FJSP is shown in Table 1. In this paper, we aim to minimize the following three objectives:

- Maximal completion time of machines (makespan).
- Workload of the most loaded machine (MW).
- Total workload of all machines (TW).

Some assumptions are put forward:

- Each operation cannot be interrupted during processing.
- Each machine can process at most one operation at any time.
- One operation cannot be processed by more than one machine simultaneously.
- Moving time between operations and setting up time of machines are negligible.
- Machines are independent of each other.
- Jobs are independent of each other.

The notations used in the definition of multiobjective FJSP in this paper are shown in Notations [25].

The mathematical model could be given as follows [25]:

Objective functions:

(1) min f1=max1≤k≤m⁡Ckmin f3=∑k=1mWk

(2) Subject  to: Cij−Cij−1≥Pijkxijk,j=2,…,ni;  ∀i,j

(3)  Chg−Cij−thgkxhgkxijk≥0 ∨Cij−Chg−tijkxhgkxijk≥0,∀i,j,h,g,k

(4)  ∑k∈Mijxijk=1

(5)  xijk=if  machine  k  is  selected  for  operation  Oij0,otherwise.

Equation (1) indicates the three optimizing objectives. Inequality (2) ensures the operation precedence constraint. Inequality (3) ensures that each machine processes only one operation at each time. Equation (4) states that one machine can be selected from the set of available machines for each operation [25]. According to whether machine *k* is selected to process step *O*_*ij*_ or not, equation (5) is used to determine the value of *x*_*ijk*_.

## 3. The Proposed Algorithm

### 3.1. Brief Introduction of NSGA-II

The nondominated sorting genetic algorithm II (NSGA-II) is a population-based multiobjective evolutionary algorithm, which is widely used in the optimization of multiobjective problems. The core procedure of NSGA-II can be briefly formulated as follows. First, using *P*_*t*_ and *U*_*t*_ presents the current parent and offspring population, respectively. The sizes of *P*_*t*_ and *U*_*t*_ are both *N*. Then a new population *R*_*t*_ = *P*_*t*_ ∪ *U*_*t*_ (of size 2*N*) is formed by combining *P*_*t*_ with *U*_*t*_. Furthermore, an operator called nondominated sorting is executed, which defines *R*_*t*_ as different nondominated levels (*rank1*,* rank2*, etc.), to choose the best *N* members as the new population named *P*_*t*+1_ for the next generation. In NSGA-II, the crowding distance is computed by a special operator, and then the solutions with larger crowding distance values are selected. More details of the NSGA-II could refer to [33].

### 3.2. The BEG-NSGA-II

#### 3.2.1. Framework

**Figure 1:**
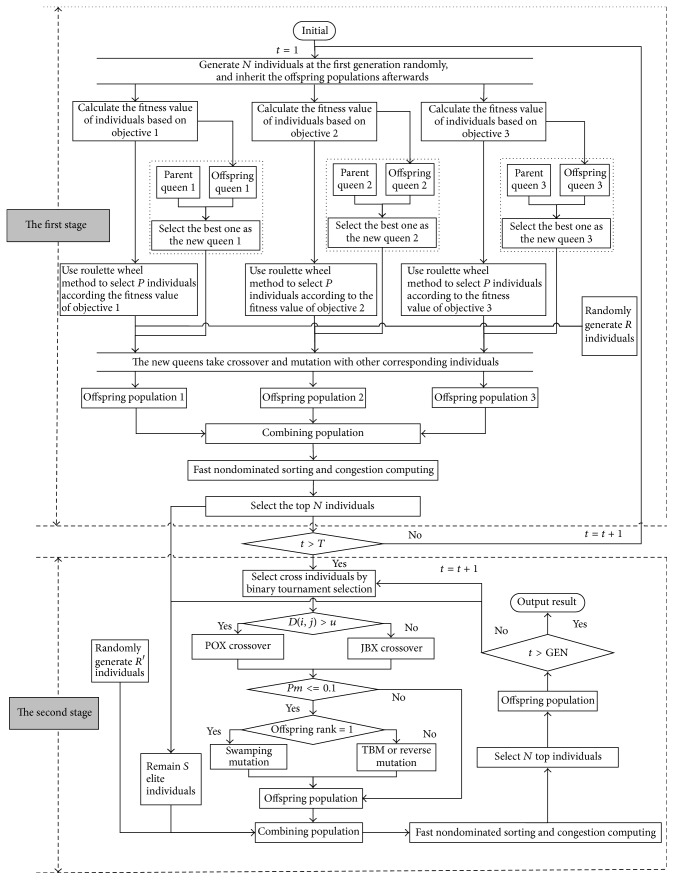
The framework of BEG-NSGA-II for MO-FJSP.

In this paper, a bee evolutionary guiding nondominated sorting genetic algorithm II (BEG-NSGA-II) with a two-stage optimization scheme is proposed for the FJSP, in which a bee evolutionary guiding scheme is presented that focus on the exploitation of solution space, and some mechanisms are used to enhance the searching ability and avoid the premature convergence. Its framework is shown in Figure 1. More details of steps are described as follows.

*The First Stage*

*Step  1.* Generate *N* individuals as the initial population randomly, and set *t* = 1. 

*Step  2.* Calculate the fitness value of individuals based on objective 1, objective 2, and objective 3; then select the best fitness individuals as the queen 1, queen 2, and queen 3 correspondingly; if *t* > 1, compare the fitness value of the parent queens and offspring queens, and select the best ones as the new queens correspondingly.

*Step  3.* Use roulette wheel method to select *P* individuals according to the fitness value of objective 1, objective 2, and objective 3.

*Step  4.* Randomly generate *R* individuals, and combine these individuals with the *P* individuals generated from* Step  3*, respectively.

*Step  5.* The new queens selected from* Step  2* take crossover and mutation with other corresponding individuals generated from* Step  4* to produce the offspring population 1, offspring population 2, and offspring population 3, respectively.

*Step  6.* Combine the three offspring populations into a combining population.

*Step  7.* Fast nondominated sorting and congestion computing.

*Step  8.* Select the top *N* individuals as the offspring population.

*Step  9.* Set *t* = *t* + 1; if *t* > *T*, continue next step; otherwise return* Step  2*, and the population is replaced by the selected *N* individuals.

*The Second Stage*

*Step  10.* Select *S* top individuals (from the top *N* individuals) as the elite individuals.

*Step  11.* Use a binary tournament selection method to select cross individuals from offspring population (i.e., the selected *N* individuals); if *D*(*i*, *j*) > *u*, use a precedence operation crossover; otherwise, use job-based crossover.

*Step  12.* If *P*_*m*_ < = 0.1 and if offspring rank = 1, then choose swapping mutation; else, choose two binding mutation or reverse mutation; else, the population does not take mutation.

*Step  13.* Randomly generate *R*′ new individuals.

*Step  14.* Combine the three-part individuals which get from* Steps  10*,* 12*, and* 13*. 

*Step  15.* Use* Step  7* to deal with the combining population to generate offspring population.

*Step  16.* If *t* > *GEN*, output the result; otherwise, *t* = *t* + 1, and return* Step  10*.

The parameters used in this algorithm are defined as follows:

At the first stage,
  (6) α=tT,P=α∗N2,R=N1−α2.
At the second stage,
  (7) α′=tGEN,
  (8) R′=N1−α′2,
  (9) χ′+S+0.5N>=N
  (10) Di,j=∑l=1Lmin⁡max⁡0,ail−ajl−1,0L,
  (11) Di,j≤u,  Precedence  operation  crossover

In (6)–(9), where *N* is the number of individuals in the initial population, *t* is the current iteration, *T* is the iteration times in the first stage, and* GEN* is the iteration times in the second stage. Equation (9) ensures that there are enough numbers of individuals to be selected in the following offspring, and we set the value of *S* as 0.3 × *N*. Equation (10) is a difference degree function which is used to compute the similarity between chromosome *i* and chromosome *j* (the two chromosomes that will be crossed), where *L* is the length of a chromosome and *a*_*il*_ denotes the *l*th gene in chromosome *i*. In (11), *u* is a parameter set as 0.7. The chromosomes tend to select the multipoint preservative crossover with the decrease of *u*.

#### 3.2.2. Chromosome Encoding and Decoding

In this paper, we use the encoding method presented by Gao et al. [12]. The FJSP problem includes two subproblems: one is operation sequence part and the other is machine assignment part. Chromosome that corresponds to the solution of the FJSP also consists of two parts. The first one is the operation sequence vector and the second one is the machine assignment vector. Two different encoding methods are used to generate the two vectors.

In terms of the operation sequence vector, the operation-based representation method is used, which is composed of the jobs' numbers. This representation uses an array of uninterrupted integers from 1 to *n*, where *n* is the total number of jobs, and each integer appears* Oni* times, where* Oni* is the total number of operations of job *i*; therefore, the length of the initial operation sequence population is equal to ∑_*i*=1_^*n*^*Oni*. By scanning the operation sequence from left to right, the *m*th occurrence of a job number expresses the *m*th operation of this job. The operation sequence vector of every initial individual of population is generated with the randomly encoding principle. By using these representation features, any permutation of the operation sequence vector can be decoded to a feasible solution.

**Figure 2:**
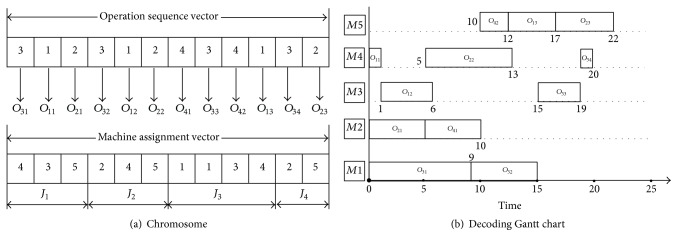
An encoding and decoding example of a chromosome of 4 × 5 FJSP.

The machine assignment vector indicates the selected machines that are assigned to the corresponding operations for all jobs. It contains *n* parts, and the length of *i*th part is* Oni*, hence the length of this vector also equals ∑_*i*=1_^*n*^*Oni*. The *i*th part of this vector expresses the machine assignment set of *i*th job. Supposing a machine set *S*_*ih*_ = {*m*_*ih*1_, *m*_*ih*2_,…, *m*_*ihCih*_} can be selected to process the *h*th operation of job *i*, a gen set {*g*_*i*1_, *g*_*i*2_,…, *g*_*ih*_,…, *g*_*iOni*_} denotes the *i*th part of machine assignment vector, and *g*_*ih*_ is an integer between 1 and *C*_*ih*_, and this means that the *h*th operation of job *i* is processed by the *g*_*ih*_th machine *m*_*ihgih*_ from *S*_*ih*_. The machine assignment vector of every initial individual of population is generated by selecting the available machine randomly for each operation of every job. An example is shown in Figure 2(a), and the operation and machine sequence are shown in it as follows: (*O*_31_, *M*_1_), (*O*_11_, *M*_4_), (*O*_21_, *M*_2_), (*O*_32_, *M*_1_), (*O*_12_, *M*_3_), (*O*_22_, *M*_4_), (*O*_41_, *M*_2_), (*O*_33_, *M*_3_), (*O*_42_, *M*_5_), (*O*_13_, *M*_5_), (*O*_34_, *M*_4_), and (*O*_23_, *M*_5_); then we can get the value of objectives of the work by referring to Table 1.

When the chromosome representation is decoded, each operation starts as soon as possible following the precedence and machine constraints [31]. A schedule generated by using this decoding method can be ensured to be active schedule [40]. The procedure of decoding is implemented as follows.

*Step  1.* Identify the machine of all operations based on the machine assignment vector.

*Step  2.* Identify the set of machines used to process every job.

*Step  3.* Identify the set of operations for every machine.

*Step  4.* Determine the allowable starting time of every operation. *AS*_*ij*_ = *C*_*i*(*j*−1)_, where *AS*_*ij*_ denotes the allowable starting time of operation *O*_*ij*_, and *C*_*i*(*j*−1)_ is the completion time of operation *O*_*i*(*j*−1)_ for the same job.

*Step  5.* Calculate the idle time of the machine of operation *O*_*ij*_, and get the idle areas [*t_*start,* t_*end], where* t_*start is the start time of these idle areas and* t_*end is the end time of these idle areas. Scanning these areas from left to right, if max (*AS*_*ij*_, *t*_start) + *t*_*ijk*_ ≤ *t*_end, the earliest starting time is *S*_*ij*_ = *t*_start; else *S*_*ij*_ = max⁡(*AS*_*ij*_, *C*_*i*(*j*−1)_). 

*Step  6.* Calculate the completion time of every operation. *C*_*ij*_ = *S*_*ij*_ + *t*_*ijk*_. 

*Step  7.* Generate the sets of starting time and completion time for every operation of every job.

By using the above procedure, a feasible schedule for the FJSP is obtained.  Figure 2 shows the examples of encoding and decoding methods, and the processing time and machine date of jobs can be seen from Table 1. This example contains 4 jobs and 5 machines. Job 1 and job 2 both have 3 operations; job 3 and job 4 contains 4 and 2 operations, respectively. Figure 2(a) shows a chromosome which contains two parts: the operation sequence vector and the machine assignment vector. The operation sequence vector is an unpartitioned permutation with repetitions of job numbers. It contains three 1s, three 2s, four 3s, and two 4s, because there are 4 jobs: job 1 contains 3 operations, job 2 contains 3 operations, job 3 contains 4 operations, and job 4 contains 2 operations. Its length is 12. The machine assignment vector consists of 4 parts because of 4 jobs. Its length is also 12. Each part presents the machines selected for the corresponding operations of job. For example, the first part contains 3 numbers which are 4, 3, and 5. Number 4 means that machine 4 is selected for operation 1 of job 1, number 3 means machine 3 is selected for operation 2 of job 1, and number 5 means machine 5 is selected for operation 3 of job 1. A Gantt chart of a schedule based on the chromosome in Figure 2(a) is shown in Figure 2(b).

#### 3.2.3. Crossover Operators

In this paper, the proposed algorithm contains two optimization stages. In order to expand the searching space and avoid premature of local optimal solutions, we use different crossover operators in these two stages.

At the first stage, a single-point crossover (SPX) or multipoint crossover (MPX) operator is selected randomly (50%) for the operation sequence vector, and a two-point crossover (TPX) operator is selected for the machine assignment vector.

The procedure of SPX is described as follows (*P*_1_ and *P*_2_ are used to denote two parents; *O*_1_ and *O*_2_ are used to denote two offspring).

*Step  1.* A random parameter *k* that meets the inequality 0 < *k* < *P* (the length of operation sequence vector) is generated to determine the position of the crossover.

*Step  2.* The elements from 1 to *k* in *P*_1_ are duplicated to *O*_1_ in the same positions; and the elements from 1 to *k* in *P*_2_ are duplicated to *O*_2_ in the same positions.

*Step  3.* Calculate the total number of each element (in this example, the total number of 1, 2, 3, and 4 is three, three, four, and two, resp.).

*Step  4.* The elements in *P*_2_ are appended to the remaining empty positions in *O*_1_ from left to right until the total number of each element in *O*_1_ equals each one in *P*_1_. The elements in *P*_1_ are appended to the remaining empty positions in *O*_2_ from left to right until the total number of each element in *O*_2_ equals each one in *P*_2_.

The procedure of MPX is described as follows (*P*_1_ and *P*_2_ are used to denote two parents; *O*_1_ and *O*_2_ are used to denote two offspring).

*Step  1.* Two random parameters *k*_1_ and *k*_2_ that meet the inequality 0 < *k*_1_ < *k*_2_ < *P* as well as *k*_1_ ≠ *k*_2_ are generated to determine the positions of crossover.

*Step  2.* The elements from *k*_1_ to *k*_2_ in *P*_1_ are appended to the leftmost positions of *O*_1_; and the elements from *k*_1_ to *k*_2_ in *P*_2_ are appended to the leftmost positions of *O*_2_. 

*Step  3.* Calculate the total number of each element (in this example, the total number of 1, 2, 3, and 4 is three, three, four, and two, resp.).

*Step  4.* The elements in *P*_2_ are appended to the remaining empty positions in *O*_1_ from left to right until the total number of each element in *O*_1_ equals each one in *P*_1_. The elements in *P*_1_ are appended to the remaining empty positions in *O*_2_ from left to right until the total number of each element in *O*_2_ equals each one in *P*_2_.

**Figure 3:**
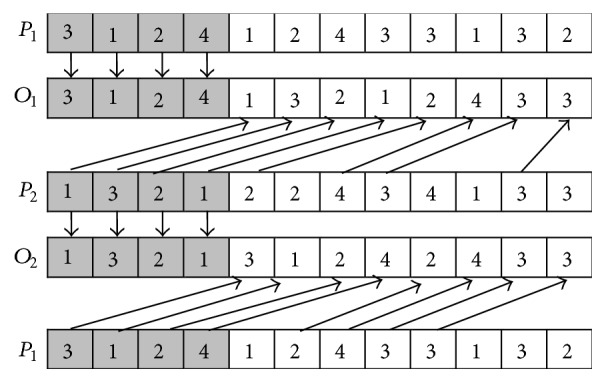
SPX crossover.

**Figure 4:**
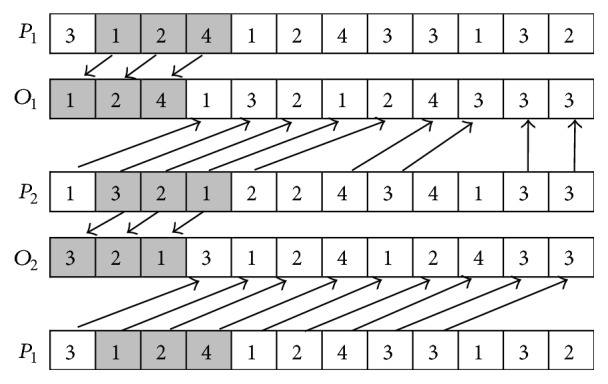
MPX crossover.

The examples of SPX and MPX are, respectively, shown in Figures 3 and 4.

For the machine assignment vector, a two-point crossover (TPX) has been adopted here as the crossover operator. The procedure of it is described as follows (*P*_1_ and *P*_2_ are used to denote two parents; *O*_1_ and *O*_2_ are used to denote two offspring).

*Step  1.* Two random parameters *k*_1_ and *k*_2_ that meet the inequality 0 < *k*_1_ < *k*_2_ < *P* as well as *k*_1_ ≠ *k*_2_ are generated to determine the positions of crossover.

*Step  2.* Append the elements between *k*_1_ and *k*_2_ positions in *P*_2_ to the same positions in *O*_1_, append the elements before *k*_1_ and after *k*_2_ positions in *P*_1_ to the same positions in *O*_1_, and use the same process to generate *O*_2_.

**Figure 5:**
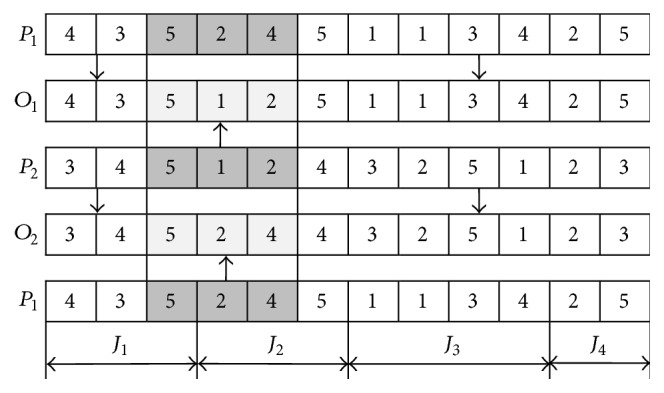
TPX crossover.

Based on the above procedure, we can obtain feasible offspring if the parents are feasible. An example of TPX crossover is shown in Figure 5.

At the second stage, a precedence operation crossover (POX) or job-based crossover (JBX), which is determined by equation (11), is selected for operation sequence vector and the TPX is also selected for machine assignment vector.

The main procedure of POX is described as follows (*P*_1_ and *P*_2_ are used to denote two parents; *O*_1_ and *O*_2_ are used to denote two offspring).

*Step  1.* The Job set is randomly divided into two subsets:* Jobset1* and* Jobset2*. 

*Step  2.* The element(s) which belong(s) to* Jobset1* in *P*_1_ is (are) appended to the same position(s) in *O*_1_ and deleted in *P*_1_; and the element(s) which belong(s) to* Jobset2* in *P*_2_ is (are) appended to the same positions in *O*_2_ and deleted in *P*_2_.

*Step  3.* Append the elements remaining in *P*_1_ to the remaining empty positions in *O*_2_ from left to right; and append the elements remaining in *P*_2_ to the remaining empty positions in *O*_1_ from left to right.

The main procedure of JBX is described as follows (*P*_1_ and *P*_2_ are used to denote two parents; *O*_1_ and *O*_2_ are used to denote two offspring).

*Step  1.* The Job set is randomly divided into two subsets:* Jobset1* and* Jobset2*. 

*Step  2.* The element(s) which belong(s) to* Jobset1* in *P*_1_ is (are) appended to the same position(s) in *O*_1_; and the element(s) which belong(s) to* Jobset2* in *P*_2_ is (are) appended to the same positions in *O*_2_. 

*Step  3.* The element(s) which belong(s) to* Jobset2* in *P*_2_ is (are) appended to the remained empty positions in *O*_1_ from left to right; and the element(s) which belong(s) to* Jobset1* in *P*_1_ is (are) appended to the remained empty positions in *O*_2_ from left to right.

**Figure 6:**
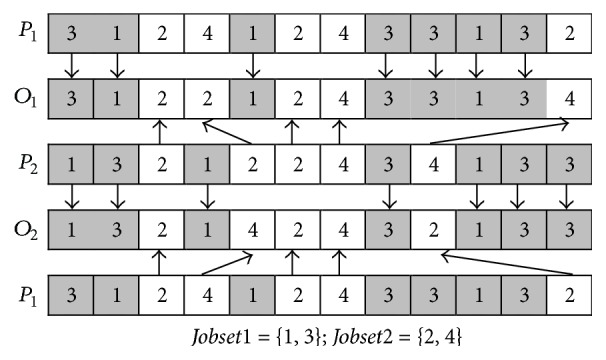
POX crossover.

**Figure 7:**
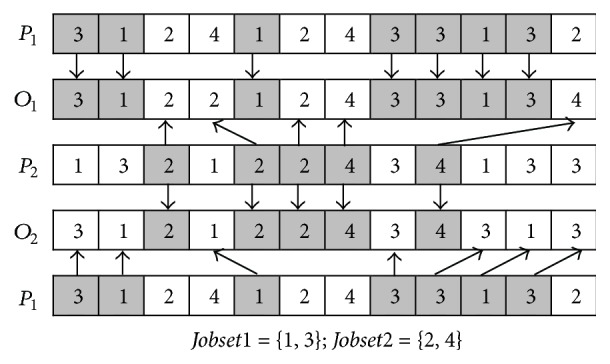
JBX crossover.

The examples of POX and JBX are, respectively, shown in Figures 6 and 7.

#### 3.2.4. Mutation Operators

In this paper, we have adopted different mutation operators at two stages for purpose of expanding the solution space as well as maintaining the good solutions.

At the first stage, a swapping mutation or reverse mutation operator is selected randomly (50%) with probability *p*_*m*_ (set as 0.1 in our algorithm) for operation sequence vector. And a multipoint mutation (MPM) operator is selected for machine assignment vector.

The main procedure of swapping mutation operator is described as follows (*P*_1_ and *O*_1_ are used to denote a parent and offspring, resp.).

*Step  1.* Randomly select two positions in *P*_1_.

*Step  2.* Swap the elements in the selected positions to generate *O*_1_. 

The main procedure of reverse mutation operator is described as follows (*P*_1_ and *O*_1_ are used to denote a parent and offspring, resp.).

*Step  1.* Randomly select two positions in *P*_1_.

*Step  2.* Reverse the numbers between the selected two positions to generate *O*_1_. 

The main procedure of MPM operator is described as follows (*P*_1_ and *O*_1_ are used to denote a parent and offspring, resp.).

*Step  1.* Randomly select *k* positions in *P*_1_ (*k* equals the half of the length of the machine assignment vector).

*Step  2.* Change the value of these selected positions according to their optional machine sets selected for process the corresponding operations. 

At the second stage, if the individual is at the forefront of Pareto, it will select the swapping mutation; otherwise choose the two binding mutation (TBM) or reverse mutation for operation sequence vector. The MPM operator is also selected for the machine assignment vector.

The main procedure of TBM is described as follows (*P*_1_ and *O*_1_ are used to denote a parent and offspring, resp.).

*Step  1.* A random parameter *m* (*m* < the length of the operation sequence vector subtract 3) is generated in *P*_1_.

*Step  2.* Exchange the elements *m* with *m* + 3 and *m* + 1 with *m* + 2 in *P*_1_ to generate *O*_1_. 

**Figure 8:**
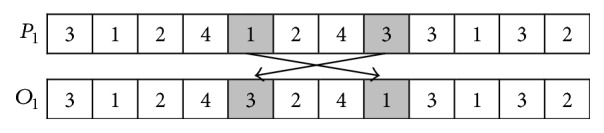
Swapping mutation operator.

**Figure 9:**
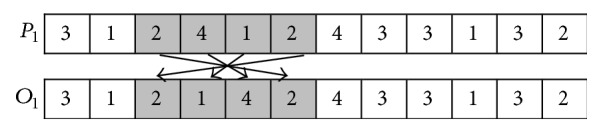
Reverse mutation operator.

**Figure 10:**
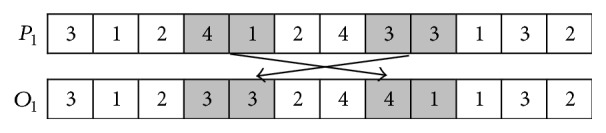
TBM mutation operator.

**Figure 11:**
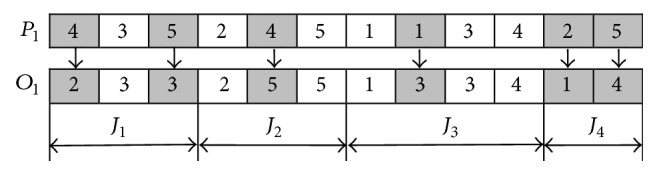
MPM operator for machine assignment vector.

The examples of swapping mutation operator, reverse mutation operator, TBM operator, and MPM operator are, respectively, shown in Figures 8, 9, 10, and 11.

## 4. Experimental Studies and Discussions

The proposed BEG-NSGA-II algorithm was coded in MATLAB R2014a and implemented on a computer configured with Intel Core i3 CPU with 2.67 GHz frequency and 4 GB RAM. Four famous benchmarks that include 53 open problems of FJSP are chosen to estimate the proposed algorithm, which were also used by many researchers to evaluate their approaches. In order to illustrate the performance of the proposed algorithm, we compare our algorithm with other state-of-the-art reported ones. The computational time used to solve these benchmarks is also compared to show the good efficiency of the proposed method. Because the computation time and implementing performance are not only affected by the algorithm itself but also affected by the computer hardware, implementing software, and coding skills, we also append the information of hardware and software, as well as the original computational time with the corresponding algorithms. All experimental simulations were run 20 times, respectively, for each problem of these benchmarks. The adopted parameters of the BEG-NSGA-II are listed in Table 2.

### 4.1. Experiment  1

The data of Experiment  1 are taken from Kacem et al. [31]. It contains 4 representative instances (problem 4 × 5, problem 8 × 8, problem 10 × 10, and problem 15 × 10). The experimental results and comparisons with other well-known algorithms are shown in Table 3 (*n* × *m* means that the problem includes *n* jobs and *m* machines; *f*_1_, *f*_2_, and *f*_3_ mean the optimization objectives of makespan, MW, and TW, resp. *T* means the average computer-independent CPU times in minute spent on each problem of these benchmarks; the symbol “—” means the time has not been given in the paper). BEG-NSGA-II denotes the proposed algorithm. The results of AL which are taken from Kacem et al. [31], PSO + SA which are taken from Xia and Wu [41], hGA which are taken from Gao et al. [24], MOGA which are taken from Wang et al. [34], hPSO which are taken from Shao et al. [42], and OO approaches which are taken from Kaplanoğlu [38] are used to make comparison with the proposed algorithm. The bolded results are the new Pareto-optimal solutions found in our algorithms.

**Figure 12:**
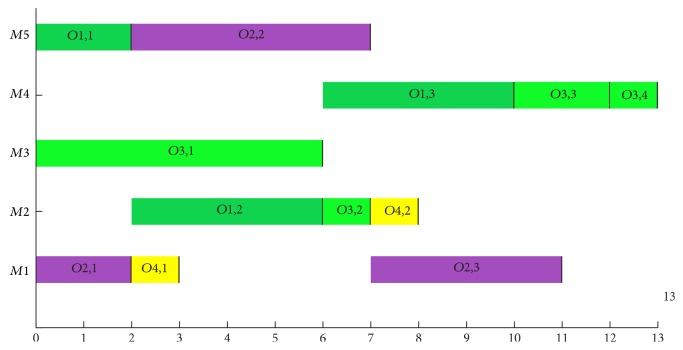
A new Pareto solution of problem 4 × 5 in Experiment  1.

For the problem 4 × 5, the proposed BEG-NSGA-II algorithm obtains not only all best solutions in these compared algorithms, but also another new Pareto-optimal solution (*f*_1_ = 13, *f*_2_ = 7, and *f*_3_ = 33). The Gantt chart of this new Pareto solution is shown in Figure 12. For the problems 8 × 8, 10 × 10, and 15 × 10, the proposed algorithm gets more Pareto-optimal solutions than other listed algorithms but hPSO, and although it obtains the same results with hPSO, it seems to consume less computation time than the hPSO does.

### 4.2. Experiment  2

**Figure 13:**
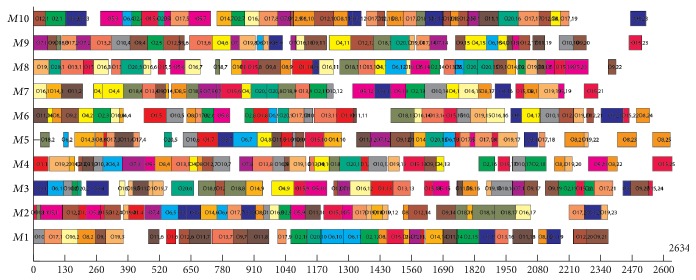
Gantt chart of a solution of problem 18a in Experiment  2.

The data of Experiment  2 are taken from Dauzère-Pérès and Paulli [10]. It contains 18 problems. The experimental results and comparisons with other well-known algorithms are shown in Table 4 (*n* × *m* means that the problem includes *n* jobs and *m* machines, *f*_1_, *f*_2_, and *f*_3_ mean the optimization objectives of makespan, MW, and TW, resp. *T* means the average computer-independent CPU times in minute spent on each problem of these benchmarks). BEG-NSGA-II denotes the proposed algorithm. The results of MOGA are adopted from Wang et al. [34]. The bolded results are the new Pareto-optimal solutions found in our algorithms. From Table 4, except the problem 01a, we can see that our algorithm can obtain some new Pareto-optimal solutions which are better for objectives *f*_2_ or *f*_3_, or both of them, but a little worse for the objective *f*_1_. For the problem 01a, our algorithm gets the same solutions as MOGA. For all problems, our algorithm consumes less computational time than MOGA.  Figure 13 illustrates the Gantt chart of the problem 18a (*f*_1_ = 2,634, *f*_2_ = 2,156, and *f*_3_ = 21,005).

### 4.3. Experiment  3

**Figure 14:**
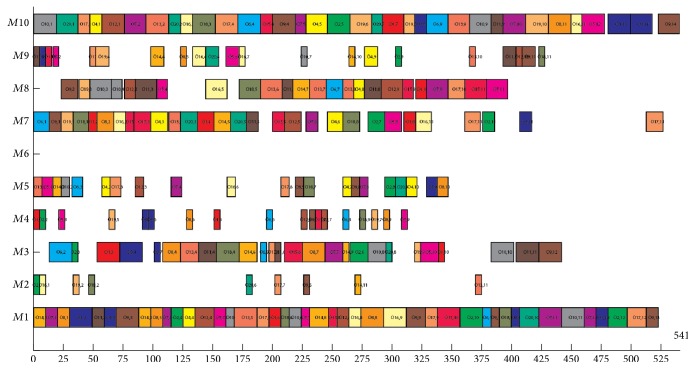
Gantt chart of a solution of problem MK08 in Experiment  3.

The data of Experiment  3 are taken from Brandimarte [9]. It contains 10 problems. The experimental results and comparisons with other well-known algorithms are shown in Table 5 (*n* × *m* means that the problem includes *n* jobs and *m* machines, *f*_1_, *f*_2_, and *f*_3_ mean the optimization objectives of makespan, MW, and TW, resp. *T* means the average computer-independent CPU times in minute spent on each problem of these benchmarks). BEG-NSGA-II denotes the proposed algorithm. The results of SM and MOGA are adopted from [26, 34]. The bolded results are the new Pareto-optimal solutions found in our algorithms. From Table 5, we can see that our algorithm can obtain some new Pareto-optimal solutions in problems MK01, MK04, MK05, MK08, and MK10 compared with SM and MOGA and the same solutions in MK2, MK3, MK6, MK7, and MK9 as MOGA. For all problems, our algorithm consumes less computational time than MOGA.  Figure 14 illustrates the Gantt chart of the problem MK08 (*f*_1_ = 541, *f*_2_ = 533, and *f*_3_ = 2,516).

### 4.4. Experiment  4

**Figure 15:**
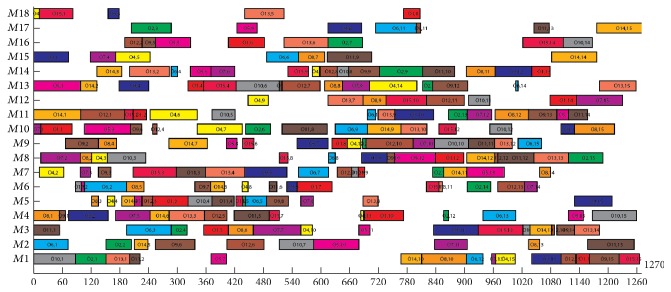
Gantt chart of problem seti5xyz in Experiment  4.

The data of Experiment  4 are taken from Barnes and Chambers [43]. It contains 21 problems. The experimental results and comparisons with other well-known algorithms are shown in Table 6 (*n* × *m* means that the problem includes *n* jobs and *m* machines, *f*_1_, *f*_2_, and *f*_3_ mean the optimization objectives of makespan, MW, and TW, resp. *T* means the average computer-independent CPU times in minute spent on each problem of these benchmarks). BEG-NSGA-II denotes the proposed algorithm. The results of HGTS and HA are adopted from [44, 45], and there are no other multiobjective optimization algorithms to compare for these problems in the up to date literatures. The bolded results are the new multiobjective optimization solution found in our algorithms. From Table 6, we can see that our algorithm can solve these problems with little worse objective *f*_1_ but simultaneously get the other two objectives *f*_2_ and *f*_3_ with less computation time.  Figure 15 illustrates the Gantt chart of the problem seti5xyz (*f*_1_ = 1270, *f*_2_ = 835, and *f*_3_ = 11,472).

### 4.5. Discussions

From the simulation results of test examples 1–3, we can see that our proposed BEG-NSGA-II could obtain the same or more different Pareto-optimal solutions for the most benchmarks of MFJSP with less computation time. That means more schemes can be chosen by the production managers when they make scheduling decisions with high efficiency. From the simulation results of test example 4, we can see that our proposed algorithm of BEG-NSGA-II works better in multiobjective optimization as well as single-objective optimization.

## 5. Conclusions and Future Studies

In this paper, in order to fully play the respective advantages of nondominated sorting genetic algorithm II (NSGA-II) algorithm and the algorithms with a two-stage optimization scheme and to overcome the disadvantages of them, we developed a bee evolutionary guiding nondominated sorting genetic algorithm II (BEG-NSGA-II) for solving the multiobjective flexible job-shop scheduling problem with the optimization objectives of minimizing the maximal completion time, the workload of the most loaded machine, and the total workload of all machines. A two-stage optimization mechanism is constructed in the optimization process. In the first stage, the NSGA-II algorithm with *T* iteration times is first used to obtain the initial population *N* which consists of three parts changing with the iteration times. In this stage, an effective local search operator is invented to extensively exploit the solution space. In the second stage, the NSGA-II algorithm with* GEN* iteration times is used to obtain the Pareto-optimal solutions, in which an updating mechanism and some useful genetic operators were employed to enhance the searching ability and avoid the premature convergence. From the simulation results, we can get the conclusions that our proposed algorithm BEG-NSGA-II could obtain more different Pareto-optimal solutions for most benchmarks of MO-FJSP with less computation time. Hence, it could provide more schemes for the production managers to choose when they make scheduling decisions. This proposed computational intelligence method (BEG-NSGA-II) could be widely used in flexible job-shop scheduling problems, especially the multiobjective optimization problems in scheduling filed.

In the future, we will concentrate on the dynamic and real-time scheduling problems which possibly include newly inserted jobs during the production process. Meanwhile, the redistribution of job operations and machine breakdowns may also be taken into consideration.

## Figures and Tables

**Table 1:** The 4 × 5 problem.

		*M*1	*M*2	*M*3	*M*4	*M*5
Job 1	*O*_1,1_	2	5	4	1	2
	*O*_1,2_	5	4	5	7	5
	*O*_1,3_	4	5	5	4	5

Job 2	*O*_2,1_	2	5	4	7	8
	*O*_2,2_	5	6	9	8	5
	*O*_2,3_	4	5	4	54	5

Job 3	*O*_3,1_	9	8	6	7	9
	*O*_3,2_	6	1	2	5	4
	*O*_3,3_	2	5	4	2	4
	*O*_3,4_	4	5	2	1	5

Job 4	*O*_4,1_	1	5	2	4	12
	*O*_4,2_	5	1	2	1	2

**Table 2:** Parameters of the BEG-NSGA-II.

Parameters	Value
Population size	100
Maximal total generation	300
Iteration times of first stage	100
Iteration times of second stage	200
Crossover probability	1
Mutation probability	0.1

**Table 3:** Results on Experiment  1.

Problem *n* × *m*	AL^a^				PSO + SA^b^				MOGA^c^				hPSO^d^				hGA^e^				OO approach^f^				BEG-NSGA-II^g^			
	*f*_1_	*f*_2_	*f*_3_	*T*	*f*_1_	*f*_2_	*f*_3_	*T*	*f*_1_	*f*_2_	*f*_3_	*T*	*f*_1_	*f*_2_	*f*_3_	*T*	*f*_1_	*f*_2_	*f*_3_	*T*	*f*_1_	*f*_2_	*f*_3_	*T*	*f*_1_	*f*_2_	*f*_3_	*T*
4 × 5	—	—	—	—	—	—	—	—	11	10	32	0.10	—	—	—	—	—	—	—	—	11	10	32	0.03	11	10	32	0.10
									11	9	34														11	9	34	
									12	8	32														12	8	32	
																									**13**	**7**	**33**	

8 × 8	16	13	75	—	15	12	75	—	15	11	81	0.16	14	12	77	0.14	15	12	75	0.01	16	13	73	0.04	14	12	77	0.12
					16	13	73		15	12	75		15	12	75						15	12	75		15	12	75	
									16	13	73		16	11	77										16	11	77	
													16	13	73										16	13	73	

10 × 10	7	5	45	—	7	6	44	—	8	5	42	0.24	7	5	43	0.20	7	5	43	0.01	8	7	41	0.05	7	5	43	0.18
	8	5	42						7	6	42		7	6	42						8	5	42		7	6	42	
	8	7	41						8	7	41		8	5	42						7	7	43		8	5	42	
									7	5	45		8	7	41										8	7	41	

15 × 10	23	11	95	—	12	11	91	—	11	11	91	1.46	11	11	91	1.36	11	11	91	0.03	13	13	91	0.09	11	11	91	0.05
	24	11	91						12	10	95		12	10	93						14	12	91		12	10	93	
									11	10	98		11	10	95										11	10	95	

^a^The computation configuration is not listed in AL.

^b^The computation configuration is not listed in PSO + SA.

^c^The CPU time on a PC with 2 GHz CPU and 2 GB of RAM memory in C++.

^d^The CPU time on a personal computer with 2 GHz CPU and 2 GB of RAM memory in C++.

^e^The CPU time on a 3.0 GHz Pentium in Delphi.

^f^The CPU time on an Intel Core i5, 2.67 GHz processor with 4 GB RAM of memory in Java.

^g^The CPU time on an Intel Core i3, 2.67 GHz processor with 4 GB RAM in MATLAB R2014a.

**Table 4:** Results on Experiment  2.

Problem (*n* × *m*)	MOGA^a^				BEG-NSGA-II^b^			
	*f*_1_	*f*_2_	*f*_3_	*T*	*f*_1_	*f*_2_	*f*_3_	*T*
01a (10 × 5)	2,568	2,505	11,137	2.04	2,568	2,505	11,137	1.91
	2,572	2,568	11,137		2,572	2,568	11,137	
	2,594	2,554	11,137		2,594	2,554	11,137	

02a (10 × 5)	2,289	2,263	11,137	2.56	**2,487**	**2,237**	**11,137**	2.13
	2,313	2,238	11,137		**3,014**	**2,234**	**11,137**	
					**2,564**	**2,236**	**11,137**	

03a (10 × 5)	2,287	2,248	11,137	2.90	**2,492**	**2,247**	**11,137**	2.5
	2,256	2,252	11,137		**2,516**	**2,234**	**11,137**	

04a (10 × 5)	2,550	2,503	11,090	2.07	**2,882**	**2,572**	**11,069**	2.03
	2,569	2,565	11,076		**2,912**	**2,552**	**11,070**	
	2,579	2,552	11,080		**2,904**	**2,626**	**11,067**	
	3,095	2,727	11,064		**3,019**	**2,665**	**11,066**	
					**2,997**	**2,523**	**11,073**	
					**2,933**	**2,688**	**11,065**	
					**2,953**	**2,668**	**11,066**	
					**2,938**	**2,606**	**11,068**	
					**2,955**	**2,503**	**11,074**	

05a (10 × 5)	2,292	2,252	11,077	2.37	**2,740**	**2,250**	**10,981**	2.12
	2,293	2,242	11,091		**2,846**	**2,212**	**10,986**	
	2,297	2,255	11,054		**2,747**	**2,229**	**10,988**	
	2,315	2,272	11,063		**2,700**	**2,255**	**10,977**	
	2,343	2,298	11,050		**2,915**	**2,231**	**10,981**	
	2,358	2,322	11,038		**2,818**	**2,208**	**10,990**	
	2,376	2,243	11,022		**2,787**	**2,350**	**10,970**	
	2,904	2,620	10,941		**2,759**	**2,320**	**10,971**	
	2,945	2,571	10,941		**2,667**	**2,237**	**10,983**	
	3,056	2,507	10,941		**2,686**	**2,277**	**10,974**	
					**2,641**	**2,332**	**10,974**	
					**2,687**	**2,221**	**11,000**	
					**2,779**	**2,237**	**10,981**	
					**2,707**	**2,299**	**10,972**	
					**2,702**	**2,233**	**10,989**	

06a (10 × 5)	2,250	2,233	11,009	3.09	**2,673**	**2,194**	**10,893**	2.86
	2,254	2,223	10,994		**2,716**	**2,194**	**10,891**	
	2,398	2,219	10,973		**2,683**	**2,245**	**10,890**	
	2,437	2,280	10,988		**2,666**	**2,248**	**10,893**	
	2,744	2,448	10,850		**2,679**	**2,229**	**10,892**	
	2,902	2,439	10,847		**2,816**	**2,186**	**10,903**	
	2,967	2,840	10,839		**2,898**	**2,186**	**10,899**	
					**2,713**	**2,234**	**10,889**	
					**2,758**	**2,209**	**10,890**	
					**2,722**	**2,229**	**10,890**	

07a (15 × 8)	2,450	2,413	16,485	7.63	**2,927**	**2,187**	**16,485**	6.21
	2,457	2,299	16,485		**2,910**	**2,213**	**16,485**	
	2,484	2,289	16,485		**2,889**	**2,264**	**16,485**	
					**2,891**	**2,219**	**16,485**	
					**2,922**	**2,190**	**16,485**	

08a (15 × 8)	2,187	2,102	16,485	8.27	**2,621**	**2,091**	**16,485**	6.35
	2,171	2,104	16,485		**2,770**	**2,089**	**16,485**	
					**2,780**	**2,080**	**16,485**	

09a (15 × 8)	2,157	2,113	16,485	10.16	**2,442**	**2,103**	**16,485**	7.26
	2,144	2,119	16,485		**2,470**	**2,074**	**16,485**	
	2,158	2,102	16,485		**2,455**	**2,081**	**16,485**	

10a (15 × 8)	2,461	2,433	16,505	7.55	**2,998**	**2,317**	**16,487**	6.43
	2,470	2,310	16,537		**3,036**	**2,363**	**16,482**	
	2,478	2,330	16,533		**3,091**	**2,491**	**16,473**	
	2,482	2,360	16,499		**2,984**	**2,319**	**16,486**	
	2,501	2,265	16,547		**3,052**	**2,377**	**16,477**	
	2,501	2,312	16,528		**3,098**	**2,491**	**16,472**	
	2,547	2,476	16,490		**3,013**	**2,293**	**16,487**	
	3,064	2,734	16,464		**3,136**	**2,457**	**16,471**	
					**3,025**	**2,377**	**16,480**	
					**3,041**	**2,351**	**16,481**	
					**2,980**	**2,258**	**16,494**	
					**3,021**	**2,319**	**16,484**	

11a (15 × 8)	2,182	2,170	16,449	10.14	**2,551**	**2,116**	**16,229**	7.25
	2,202	2,114	16,476		**2,599**	**2,131**	**16,224**	
	2,210	2,113	16,442		**2,612**	**2,146**	**16,222**	
	2,337	2,185	16,377		**2,573**	**2,058**	**16,231**	
	2,874	2,389	16,247		**2,753**	**2,186**	**16,217**	
	2,894	2,330	16,247		**2,628**	**2,102**	**16,224**	
	2,962	2,312	16,247		**2,702**	**2,098**	**16,223**	
					**2,658**	**2,089**	**16,224**	
					**2,555**	**2,111**	**16,229**	
					**2,664**	**2,063**	**16,228**	

12a (15 × 8)	2,161	2,107	16,295	11.92	**2,624**	**2,049**	**16,055**	8.22
	2,168	2,130	16,220		**2,620**	**2,022**	**16,068**	
	2,191	2,084	16,355		**2,587**	**2,035**	**16,065**	
	2,210	2,103	16,331		**2,614**	**2,057**	**16,051**	
	2,315	2,125	16,292		**2,585**	**2,043**	**16,061**	
	2,366	2,105	16,237		**2,659**	**2,038**	**16,055**	
	2,493	2,297	16,124		**2,617**	**2,024**	**16,065**	
	2,631	2,309	16,112		**2,575**	**2,057**	**16,057**	
	2,637	2,303	16,113		**2,587**	**2,050**	**16,059**	
	2,683	2,397	16,104		**2,582**	**2,098**	**16,052**	

13a (20 × 10)					**2,595**	**2,036**	**16,059**	
					**2,566**	**2,044**	**16,061**	
	2,408	2,326	21,610	23.99	**2,894**	**2,277**	**21,610**	10.27
					**2,928**	**2,233**	**21,610**	
					**2,896**	**2,244**	**21,610**	
					**2,877**	**2,282**	**21,610**	

14a (20 × 10)	2,340	2,251	21,610	29.05	**2,649**	**2,186**	**21,610**	12.39
	2,334	2,258	21,610		**2,664**	**2,183**	**21,610**	
					**2,641**	**2,207**	**21,610**	
					**2,738**	**2,182**	**21,610**	

15a (20 × 10)	2,285	2,247	21,610	33.29	**2,627**	**2,216**	**21,610**	13.60
	2,287	2,218	21,610		**2,681**	**2,196**	**21,610**	
					**2,645**	**2,215**	**21,610**	
					**2,652**	**2,201**	**21,610**	
					**2,668**	**2,197**	**21,610**	
					**2,682**	**2,178**	**21,610**	

16a (20 × 10)	2,447	2,354	21,602	21.52	**2,965**	**2,279**	**21,534**	11.48
	2,450	2,380	21,590		**3,158**	**2,303**	**21,504**	
	2,487	2,454	21,584		**3,184**	**2,400**	**21,490**	
	2,492	2,417	21,576		**3,039**	**2,279**	**21,523**	
	2,540	2,396	21,547		**3,050**	**2,318**	**21,507**	
	2,550	2,492	21,545		**3,071**	**2,286**	**21,513**	
	2,568	2,428	21,540		**3,275**	**2,281**	**21,511**	
	3,013	2,588	21,478		**3,105**	**2,352**	**21,496**	
	3,106	2,548	21,478		**3,175**	**2,344**	**21,497**	
					**3,180**	**2,293**	**21,507**	
					**3,130**	**2,312**	**21,503**	
					**3,034**	**2,352**	**21,503**	

17a (20 × 10)	2,322	2,240	21,433	28.47	**2,806**	**2,195**	**21,096**	13.92
	2,322	2,280	21,362		**2,750**	**2,165**	**21,120**	
	2,323	2,238	21,454		**2,791**	**2,141**	**21,114**	
	2,343	2,224	21,420		**2,716**	**2,157**	**21,129**	
	2,480	2,285	21,344		**2,849**	**2,148**	**21,107**	
	2,528	2,231	21,313		**2,769**	**2,166**	**21,111**	
	2,789	2,448	21,198		**2,856**	**2,183**	**21,098**	
	2,808	2,303	21,200		**2,773**	**2,145**	**21,111**	
	2,816	2,370	21,197		**2,856**	**2,142**	**21,105**	
					**2,803**	**2,203**	**21,094**	
					**2,759**	**2,186**	**21,106**	
					**2,789**	**2,176**	**21,101**	
					**2,772**	**2,202**	**21,101**	

18a (20 × 10)	2,267	2,235	21,483	33.01	**2,634**	**2,156**	**21,005**	16.23
	2,269	2,206	21,408		**2,642**	**2,126**	**21,006**	
	2,320	2,208	21,354		**2,638**	**2,185**	**20,999**	
	2,437	2,221	21,311		**2,638**	**2,148**	**21,002**	
	2,531	2,310	21,285		**2,674**	**2,148**	**21,000**	
	2,545	2,305	21,282		**2,644**	**2,145**	**21,002**	
					**2,651**	**2,140**	**21,005**	
					**2,641**	**2,157**	**21,000**	
					**2,664**	**2,145**	**21,001**	
					**2,621**	**2,181**	**21,002**	
					**2,659**	**2,140**	**21,003**	
					**2,661**	**2,137**	**21,004**	
					**2,651**	**2,181**	**20,999**	
					**2,669**	**2,126**	**21,005**	
					**2,708**	**2,125**	**21,009**	

^a^The CPU time on a PC with 2 GHz CPU and 2 GB of RAM memory in C++.

^b^The CPU time on an Intel Core i3, 2.67 GHz processor with 4 GB RAM in MATLAB R2014a.

**Table 5:** Results on Experiment  3.

Problem (*n* × *m*)	SM^a^				MOGA^b^				BEG-NSGA-II^b^			
	*f*_1_	*f*_2_	*f*_3_	*T*	*f*_1_	*f*_2_	*f*_3_	*T*	*f*_1_	*f*_2_	*f*_3_	*T*
MK01 (10 × 6)	42	42	162	4.78	42	39	158	0.49	42	39	158	0.36
					44	40	154		44	40	154	
					43	40	155		43	40	155	
					40	36	169		40	36	169	
									**47**	**36**	**167**	
									**47**	**37**	**165**	
									**49**	**37**	**164**	
									**50**	**38**	**162**	

MK02 (10 × 6)	28	28	155	3.02	26	26	151	0.75	26	26	151	0.70
					27	27	146		27	27	146	
					29	27	145		29	27	145	
					29	29	143		29	29	143	
					31	31	141		31	31	141	
					33	33	140		33	33	140	

MK03 (15 × 8)	204	204	852	26.14	204	199	855	4.75	204	199	855	3.66
					204	144	871		204	144	871	
					204	135	882		204	135	882	
					204	133	884		204	133	884	
					213	199	850		213	199	850	
					214	210	849		214	210	849	
					221	199	847		221	199	847	
					222	199	848		222	199	848	
					231	188	848		231	188	848	
					230	177	848		230	177	848	

MK04 (15 × 8)	68	67	352	17.74	66	63	345	1.76	66	63	345	1.63
					65	63	362		65	63	362	
					63	61	371		63	61	371	
					62	61	373		60	59	390	
					61	60	382		74	54	349	
					60	59	390		74	55	348	
					73	55	350		**72**	**72**	**342**	
					74	54	349		**78**	**78**	**339**	
					74	55	348		**72**	**66**	**348**	
					90	76	331		**73**	**72**	**343**	

MK05 (15 × 4)	177	177	702	8.26	173	173	683	2.34	173	173	683	1.96
					175	175	682		175	175	682	
					183	183	677		183	183	677	
					185	185	676		179	179	679	
					179	179	679		**199**	**199**	**674**	
									**193**	**193**	**675**	
									**183**	**183**	**677**	

MK06 (10 × 15)	67	431	18.79		62	55	424	1.93	62	55	424	1.85
					65	54	417		65	54	417	
					60	58	441		60	58	441	
					62	60	440		62	60	440	
					76	60	362		76	60	362	
					76	74	356		76	74	356	
					78	60	361		78	60	361	
					73	72	360		73	72	360	
					72	72	361		72	72	361	
					100	90	330		100	90	330	

MK07 (20 × 5)	150	150	717	5.68	139	139	693	4.92	139	139	693	3.12
					140	138	686		140	138	686	
					144	144	673		144	144	673	
					151	151	667		151	151	667	
					157	157	662		157	157	662	
					162	162	659		162	162	659	
					166	166	657		166	166	657	

MK08 (20 × 10)	523	523	2,524	67.67	523	515	2,524	12.04	523	515	2,524	8.34
					523	497	2,534		523	497	2,534	
					524	524	2,519		524	524	2,519	
					578	578	2,489		578	578	2,489	
					587	587	2,484		587	587	2,484	
									**541**	**533**	**2,516**	
									**542**	**542**	**2,511**	
									**560**	**560**	**2,505**	

MK09 (20 × 10)	311	299	2,374	77.76	311	299	2,290	19.48	311	299	2,290	10.15
					310	299	3514		310	299	3514	
					311	301	2,287		311	301	2,287	
					314	299	2,315		314	299	2,315	
					315	299	2,283		315	299	2,283	
					332	302	2,265		332	302	2,265	
					329	301	2,266		329	301	2,266	
					328	308	2,259		328	308	2,259	
					325	299	2,275		325	299	2,275	

MK10 (20 × 15)	227	221	1,989	122.52	224	219	1,980	17.87	224	219	1,980	12.38
					225	211	1,976		235	225	1,895	
					233	214	1,919		240	215	1,905	
					235	218	1,897		246	215	1,896	
					235	225	1,895		252	224	1,884	
					240	215	1,905		256	211	1,919	
					240	216	1,888		260	244	1,869	
					242	214	1,913		266	254	1,864	
					246	215	1,896		**246**	**206**	**1,938**	
					252	224	1,884		**246**	**204**	**1,948**	
					256	211	1,919		**246**	**205**	**1,940**	
					260	244	1,869		**251**	**202**	**1,948**	
					266	254	1,864		**253**	**207**	**1,930**	
					268	264	1,858		**249**	**203**	**1,947**	
					276	256	1,857		**255**	**202**	**1,943**	
					281	268	1,854		**260**	**210**	**1,926**	
					217	207	2,064		**250**	**205**	**1,935**	
					214	204	2,082		**254**	**203**	**1,942**	
									**244**	**207**	**1,935**	

^a^The CPU time on a Pentium IV 2.4 GHz processor with 1.0 GB of RAM memory in MATLAB.

^b^The CPU time on a PC with 2 GHz CPU and 2 GB of RAM memory in C++.

^c^The CPU time on an Intel Core i3, 2.67 GHz processor with 4 GB RAM in MATLAB R2014a.

**Table 6:** Results on Experiment  4.

Problem (*n* × *m*)	HGTS^a^		HA^b^		BEG-NSGA-II^c^			
	*f*_1_	*T*	*f*_1_	*T*	*f*_1_	*f*_2_	*f*_3_	*T*
mt10c1 (10 × 11)	927	0.22	927	0.20	**1,092**	**631**	**5,109**	0.18
mt10cc (10 × 12)	908	0.22	908	0.16	**1,005**	**631**	**5,109**	0.20
mt10x (10 × 11)	918	0.25	918	0.18	**1,117**	**556**	**5,109**	0.18
mt10xx (10 × 12)	918	0.20	918	0.18	**1,117**	**556**	**5,109**	0.18
mt10xxx (10 × 13)	918	0.20	918	0.18	**1,100**	**556**	**5,109**	0.19
mt10xy (10 × 12)	905	0.22	905	0.19	**1,061**	**548**	**5,109**	0.21
mt10xyz (10 × 13)	847	0.30	847	0.16	**925**	**534**	**5,109**	0.23
setb4c9 (15 × 11)	914	0.27	914	0.26	**1,035**	**857**	**7,727**	0.25
setb4cc (15 × 12)	907	0.25	907	0.25	**1,023**	**857**	**7,727**	0.24
setb4x (15 × 11)	925	0.25	925	0.21	**1,030**	**846**	**7,727**	0.20
setb4xx (15 × 12)	925	0.23	925	0.09	**1,030**	**846**	**7,727**	0.10
setb4xxx (15 × 13)	925	0.25	925	0.15	**1,033**	**846**	**7,727**	0.16
setb4xy (15 × 12)	910	0.32	910	0.20	**1,020**	**845**	**7,727**	0.22
setb4xyz (15 × 13)	905	0.25	905	0.24	**1,011**	**838**	**7,727**	0.23
seti5c12 (15 × 16)	1,170	0.68	1,170	0.52	**1,319**	**1,027**	**11,472**	0.48
seti5cc (15 × 17)	1,136	0.57	1,136	0.28	**1,301**	**888**	**11,472**	0.29
seti5x (15 × 16)	1,199	0.63	1,198	0.46	**1,368**	**938**	**11,472**	0.52
seti5xx (15 × 17)	1,197	0.57	1,197	0.48	**1,346**	**938**	**11,472**	0.55
seti5xxx (15 × 18)	1,197	0.52	1,197	0.32	**1,346**	**938**	**11,472**	0.37
seti5xy (15 × 17)	1,136	0.57	1,136	0.29	**1,301**	**888**	**11,472**	0.27
seti5xyz (15 × 18)	1,125	0.72	1,125	0.55	**1,270**	**835**	**11,472**	0.61

^a^The CPU time on a Xeon E5520 processor with 24 GB of RAM memory in C++.

^b^The CPU time on an Intel 2.0 GHz Core (TM) 2 Duo processor with 8.0 GB of RAM memory in C++.

^c^The CPU time on an Intel Core i3, 2.67 GHz processor with 4 GB RAM in MATLAB R2014a.

## Notations

*n*: Total number of jobs
*m*: Total number of machines
*n*_*i*_: Total number of operations of job *i*
*O*_*ij*_: The *j*th operation of job *i*
*M*_*ij*_: The set of available machines for the operation
*P*_*ijk*_: Processing time of *O*_*ij*_ on machine *k*
*t*_*ijk*_: Starting time of operation *O*_*ij*_ on machine *k*
*C*_*ij*_: Completion time of the operation *O*_*ij*_
*i*, *h*: Index of jobs, *i*, *h* = 1,2,…, *n*
*k*: Index of machines, *k* = 1,2 …, *m*
*j*, *g*: Index of operation sequence, *j*, *g* = 1,2 …, *n*_*i*_
*C*_*k*_: The completion time of *M*_*k*_
*W*_*k*_: The workload of *M*_*k*_.
